# Knowledge, attitudes and practices about malaria in Cabo Verde: a country in the pre-elimination context

**DOI:** 10.1186/s12889-019-7130-5

**Published:** 2019-07-01

**Authors:** Adilson José DePina, Abdoulaye Kane Dia, Antonieta de Ascenção Soares Martins, Maria Celina Ferreira, António Lima Moreira, Silvania Veiga Leal, Cecílio Mendes Pires, Jaelsa Mira Gomes Moreira, Maria Filomena Tavares, Aires Januário Fernandes da Moura, José Manuel Pereira, Ousmane Faye, Ibrahima Seck, El Hadji Amadou Niang

**Affiliations:** 1 Ecole Doctorale des Sciences de la Vie, de la Santé et de l’Environnement (ED-SEV), Université Cheikh Anta Diop (UCAD) de Dakar, Dakar, Sénégal; 2 Programa de Pré-Eliminação do Paludismo, CCS-SIDA. Ministério da Saúde e da Segurança Social, Avenida Cidade Lisboa, Prêdio Bô Casa, 1° Andar; CP, 855 Praia, Cabo Verde; 3 Laboratoire d’Ecologie Vectorielle et Parasitaire, Faculté des Sciences et Techniques, Université Cheikh Anta Diop (UCAD) de Dakar, Dakar, Sénégal; 4 Faculdade de Ciências e Tecnologia, Universidade de Cabo Verde, Praia, Cabo Verde; 5 Unidade de Seguimento e Avaliação, CCS-SIDA. Ministério da Saúde e da Segurança Social, Praia, Cabo Verde; 6 Programa Nacional de Luta contra o Paludismo, Ministério da Saúde e da Segurança Social, Praia, Cabo Verde; 7 Laboratório de Entomologia Médica, Instituto Nacional de Saúde Pública, Praia, Cabo Verde; 8 Unidade de Unidade Microbiologia Médica; Departamento de Virologia, Instituto de Higiene e Medicina Tropical, Lisbon, Portugal; 9 Laboratório de Análises Clínica, Hospital Regional de Santiago Norte, Assomada, Cabo Verde; 10 Delegacia de Saúde da Praia, Praia, Cabo Verde; 11 Rede Nacional de Laboratório, Ministério da Saúde e da Segurança Social, Praia, Cabo Verde; 12 Unidade da Unidade de Ciências da Natureza, da Vida e do Ambiente. Universidade Jean Piaget de Cabo Verde, Praia, Cabo Verde; 13 Laboratório de Engenharia Civil, Praia, Cabo Verde; 14 Institut de Santé et Développement, Université Cheikh Anta Diop (UCAD) de Dakar, Dakar, Sénégal; 15 Aix Marseille Univ, IRD, AP-HM, MEPHI, IHU-Méditerranée Infection, Marseille, France

**Keywords:** KAP, Knowledge, Attitudes, Practices, Malaria elimination, Cape Verde

## Abstract

**Background:**

Malaria in Cape Verde is unstable, with a sporadic and seasonal transmission of low endemicity. In this sense, the community perceptions regarding malaria transmission, their attitudes and practices against the disease are very important to understand and to better develop the best strategical policies to achieve malaria elimination goal. This study aim to assess the knowledge, attitudes and practices (KAP) of Cape Verdean population about malaria, a country in the elimination step of disease.

**Methods:**

A cross-sectional malaria KAP Survey was performed at the household level. A structured open questionnaire was developed and applied to residents of randomly selected households from 5 islands and 15 municipalities in Cape Verde. Correlation analyses were performed using a logistic regression model to determine the factors that are associated with the complete knowledge of the population about malaria.

**Results:**

A total of 1953 fully completed questionnaires were analysed, with majority of questionnaires administered in Santiago island (68.3%), mainly in the capital city of Praia, 38.43%. About 88% of the population knew the correct form of transmission, 96% had knowledge that the entire population is at risk of malaria and identified the main symptoms. Regarding the attitudes, 58% seek treatment atthe nearest health structure upon the apparition of the symptoms, 64% in the first 24 h and 88% within the first 48 h. More than 97% have heard about mosquito nets but only 19% used it. In practice, 53% use coils, 45% rely on household sprays and 43% have benefited from IRS. About 90% received information about malaria from media, mainly the TV and the radio (83 and 43%, respectively). In summary, 54% of the population has complete knowledge of the disease.

**Conclusion:**

The population of Cape Verde has a high level of knowledge about malaria, including its transmission, main symptoms and preventive and control measures. However, some gaps and misunderstandings have been noticed and contribute to the insufficient community involvement in actions against malaria. Therefore, is necessary to increase the knowledge of the population, leading to their full ownership and participation in community actions to contribute to the malaria elimination in the country.

**Electronic supplementary material:**

The online version of this article (10.1186/s12889-019-7130-5) contains supplementary material, which is available to authorized users.

## Background

Despite being a preventable and curable disease, malaria remains a major public health problem with a huge economic burden. In 2017, approximately 219 million cases responsible for about 451,000 malaria deaths were recorded worldwide. The African region accounting for more than 90% cases and 93% of all deaths remains the most affected of all the World Health Organisation (WHO) regions [1].

Currently, of the 91 countries with ongoing malaria transmission, 14 are located in sub-Saharan Africa and account, with India, for more than 80% of the global malaria burden. Therefore, the WHO has rolled out a significant malaria control effort worldwide to reduce the disease incidence and to drive toward malaria elimination where feasible. Consequently, the estimated global malaria incidence has decreased by 18%, from 72 to 59 cases per 1000 populations at risk from 2010 to 2017 [1]. In 2017, Paraguay and Uzbekistan were certified malaria-free by the WHO, while 46 countries reported less than 10,000 malaria cases, and 26 (including the Cabo Verde) having the potential to eliminate malaria by 2020.

Historically, malaria has been officially reported in the Archipelago of Cape Verde since the sixteenth century when the islands were settled [2]. During that time, the disease was endemic and seasonal with the risk of outbreaks, especially during years of high rainfall records, with together high migrants’ flux from malaria hyperendemic regions, namely São Tomé and Príncipe, Angola or Guinea- Bissau [3].

Therefore, until the 1940s, malaria represented a serious public health problem in Cape Verde, with severe epidemics affecting annually more than 10,000 persons and responsible for more than 200 deaths [4]. The implementation of semi-annual campaigns of Indoor Residual Spray (IRS) in all the lived islands between 1967 and 1972 then between 1983 and 1985, have resulted to the elimination of the disease in the country. As such the country has been declared free of malaria twice. But the diseases resurged respectively 5 and 3 years afterwards the first and the second elimination periods.

Since 1987, started the third period characterized by recurrent malaria outbreaks, mainly in the island of Santiago where all the indigenous cases occurred up to 2003 with the report of local malaria transmission in the island of Boavista [5]. However, conversely to autochthonous cases, imported cases have been reported in almost the entire national territory [6].

Nowadays, in Cape Verde malaria is seasonal and unstable, with the sporadic transmission of low endemicity. The transmission varies annually with a peak during the rainy season, responsible for the temporal fluctuation of malaria morbidity as observed in areas of risk of malaria transmission across the Archipelago. In the islands of Santiago and Boavista, where all the indigenous cases were recorded recently, malaria affects mostly young men of an average age of 20 years, while children under five and the pregnant women seem to be less impacted. Noteworthily, no malaria infection has been diagnosed in a pregnant women in 2017 [6].

With the advances in the fight against malaria in Cape Verde, the archipelago has been designated by the WHO as one of the countries with the potential to eliminate malaria by 2020. Despite the significant successes made so far against the disease, including its elimination twice from the archipelago in the past, it is worth noting the failure to consolidate and maintain the malaria-free status. Thegoal of achieving zero local transmission by 2020, has been challenged by the recent malaria outbreak with 423 indigenous cases recorded in 2017 [7].

Considering the complexity and challenges for malaria elimination, there is no doubt that all malaria preventive and control interventions should be community-based and requires full ownership from the affected local populations. However, their appropriation of any control programme, as well as their willingness to accept interventions, are influenced by their knowledge and perceptions about diseases and interventions. Moreover, the local population daily behaviour also highly crucial is determined by several other factors related to their level of knowledge about malaria [8].

Health education to enhance and increase knowledge and awareness in the population, through community engagement leads to good participation in the interventions for malaria control and elimination. Whereby in an area with low malaria cases, as Cape Verde, the community diagnosis about the social and human behavioural aspects, becomes essential before all intervention [8]. Findings from KAP surveys can be formative to guide malaria in vector control, case management, implementation of behavioural interventions, promotion of health-seeking behaviour and ultimately enhance community participation and engagement [9–14]. In Cape Verde archipelago, with islands where locally acquired malaria cases have not been recorded for decades [6], this malaria KAP survey provide key elements not only to the NMCP in the elimination context, to empower local populations and enhance their ownership and responsibility, but also to promote the application of action-oriented and participatory approaches. Therefore, the objective of this study was to evaluate the KAP about malaria among Cape Verdean population and to provide insights on effective malaria actions to get back on the track of the malaria elimination goal in the country.

## Methods

### The country

Cabo Verde is an archipelago of ten volcanic, of which nine islands are inhabited and islands and several islets. The islands are divided into two groups located toward the western coast of the African continent.

**Fig. 1:**
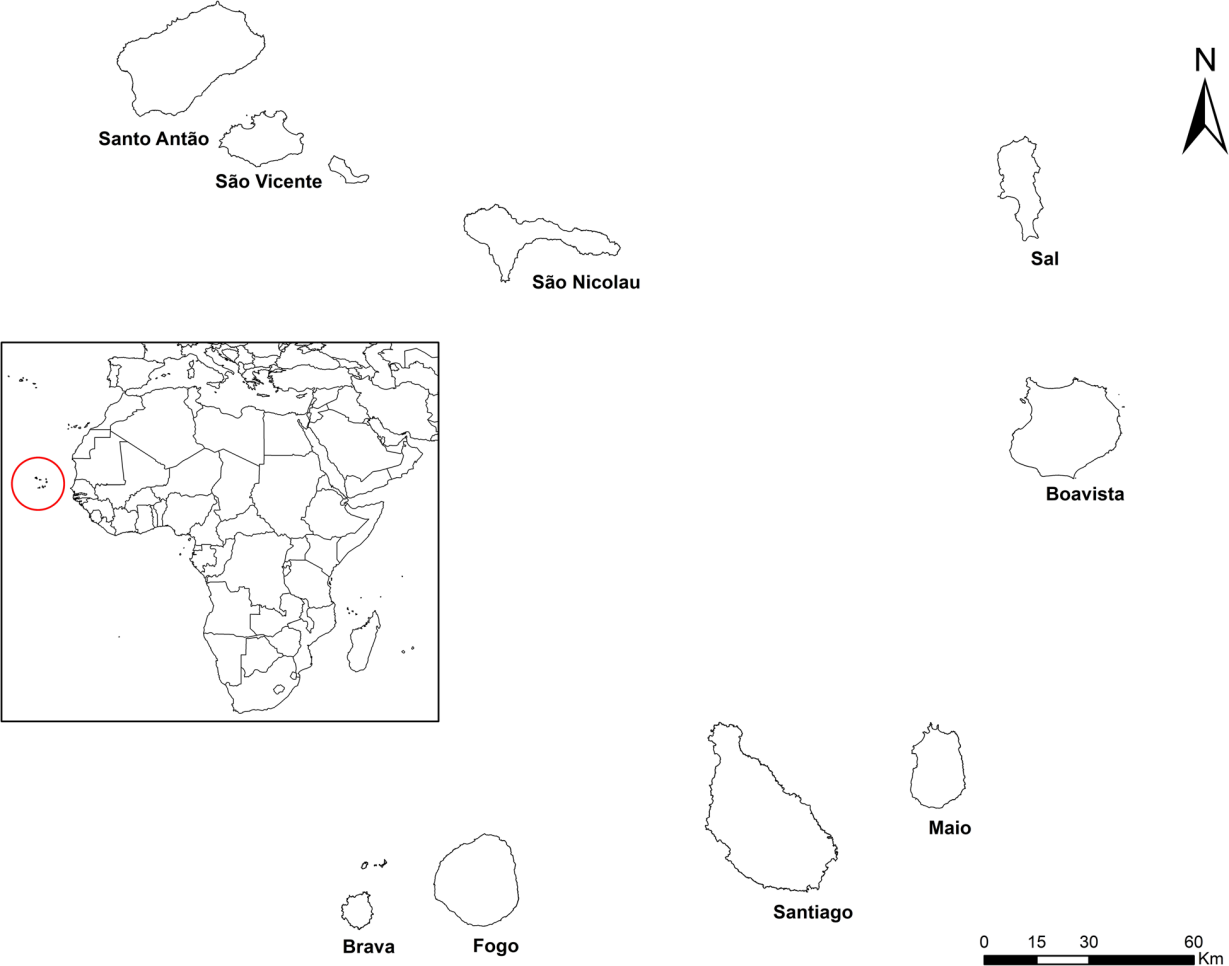
Location of the Cape Verde islands

The country covers an area of 4033 km^2^ and is located between the 17° 12 ‘and 14° 48’ latitudes and 22° 44′ and 25° 22′ longitudes. The islands are relatively sparse, and the relief is mostly rocky, with only about 10% of the soils suitable for agriculture (Fig. 1).

According to the 2010 National Census, the population of Cabo Verde has increased by 1.23%, from 434,625 to 491,575 inhabitants between 2000 and 2010. About 62% of the population live in urban areas with a slight predominance of women. Overall, children under 15 years of age represent 31.7% of the general population and the average age increased from 18 to 22 years. The average household size is 4.2 persons per household. Despite being a country of emigrants, the country has also become, in recent decades, an important destination for immigrants, mainly from African countries, but also from Europe and Asia. Indeed, in 2010, 2.9% (14.373) of the country residents were migrants [15].

### The KAP survey

A cross-sectional malaria KAP Survey was performed between April to May 2017 at the household-level, using semi-structured questionnaire (available in Additional file 1) adapted and administrated to residents of randomly selected households from 15 municipalities in five out of the nine islands of the country, namely, São Vicente, Boavista, Maio, Santiago and Fogo. The interviews were carried out by 65 municipal health technicians, trained for this purpose, and supervised by 8 coordinators.

A random stratification method was used to choose the study participants to obtain a representative sample. Samples were chosen based on the characteristics of the household, and interview of the head of the household or the responsible adult, using a standard questionnaire. The sample size was calculated according to the number of households and the average number of individuals per family in each municipality as recorded from the last population census carried out in Cape Verde [9]. Therefore, samples were selected based on the population proportion with absolute precision (*d*) of 2.5%, a confidence interval of 95% (*z = 1.96*) and *p-value* set at 0.5. Given the objectives of the study, the samples size was calculated taking into account the target population, the resident population and the number of family dwellings occupied as habitual residence. Thehe quality of questionnaire data was monitored daily.

#### Selection of variables

The questionnaire was structured to take approximately 25 min and included open and closed questions, structured in five distinct parts: (i) socio-economic and demographic characteristics, (ii) assessment of knowledge, (iii) attitudes and (iv) and preventive practices related to malaria, including the use of mosquito nets, and (v), a final section concerned the source of communication and information about the control of malaria in the community.

After answering all the questions, the participant was asked to guide the field team inside and around their home, with the purpose of completing the checklist. At the end of the interview, each interviewer provides the participant with the summary of the main information collected for review for validation by respondents.

#### Data analysis

A total of 1953 semi-structured interviews were conducted and the data collected were analysed using the SPSS 21.0 software version. The results were presented as descriptive analysis, in frequencies and percentage for categorical variables to determine the level of knowledge, the main attitudes and practices of the population. The qualitative data were analysed using the documentary thematic analysis in accord with the contents of the interviews.

Additionally, the population’s complete knowledge about malaria was further analysed, to determine the percentage of respondents who simultaneously knows the form of transmission of the disease, one of the main symptoms of the disease and a preventive measure against the disease. Subsequently, a detailed analysis was carried out through logistic regression, where the variables were selected based on the significant association in relation to the complete knowledge of malaria, taking into account the relevance to the research question [11]. For the logistic regression analysis, thus selected variables were dichotomized into two categories with presence of certain characters/conditions = 1 and absence = 0. A univariate analysis was conducted with the outcome variable to calculate crude odds ratios and a multivariate analysis, selected variables were included into the model to calculate the adjusted odds ratio and a Statistical significance with *p*-value ≤0.05 [16, 17].

#### Ethical clearance

The study protocol was approved by the National Ethics and Health Research Committee (CNEPS), in accordance with resolution 18/2017. Participation was voluntary, and no personal identification document was recorded during the data collection. All data was stored in a protected file with access to information limited to a small team in charge of piloting the study. The confidentiality of the data provided by the respondents was highlighted as one of the formative modules in the training of the interviewers.

The purpose, procedures, duration of the study and related risks and benefits were all clearly explained to participants prior to their participation in any of the steps in the data collection process. Before signing the consent form for participation, participants had the opportunity to ask clarifying questions about the study. Participants who agreed to take part in the study were invited to describe the study in order to clarify any misconception and to ascertain whether the objectives were assimilated. The speakers gave explanations about the possibility of withdrawing from interviews or group discussions at any time. The terms of consent for this study will be destroyed as soon as all analyses of the results are completed.

## Results

### Descriptive analysis

A total of 1.953 fully completed questionnaires from the interviewed families were used in the final analysis of the KAP study in the country. Majority of the questionnaires were carried out in the capital, Praia (749), followed by São Vicente (15.9%), Santa Catarina (8.4), and Santa Cruz (5.6%) (Table 1).

**Table 1:** Number of interviews by municipalities and island

Island	Municipality	Percentage (%)	n
São Vicente	São Vicente	15.9	310
Boa Vista	Boa Vista	3.6	71
Maio	Maio	2.3	44
Santiago	Tarrafal	4.2	83
	Santa Catarina	8.4	165
	Santa Cruz	5.6	110
	Praia	38.4	749
	São Domingos	1.1	22
	São Miguel	5.5	108
	São Salvador do Mundo	1.5	29
	São Lourenço Orgãos	1.6	32
	Ra Grande Santiago	2.0	40
Fogo	Mosteiros	2.5	48
	São Filipe	5.4	106
	Santa Catarina	1.8	36
	TOTAL	100.0	1953

The average age of respondents was 44 years, ranging from 15 to 64 years. The majority of participants were female (72.0%), youth (23.8%, aged between 25 to 34 years), single (51.1%) and with primary or secondary school level (47.7 and 32.9%, respectively). At least one person works with a fixed salary in about 43.2% of the households. However, in some cases, in 33,7% and 12,5% of respondents, has 02, 03 or more people, respectively, are employed and earn a salary (Table 2).

**Table 2:** Characteristics of the KAP study population in Cabo Verde 2017

Characteristics	Participation	
	Percentage (%)	n
Age range		
≤ 29	25.3	495
30–39	21.1	412
≥ 40	53.6	1046
Sex		
Male	28.0	546
Female	72.0	1407
Civil status		
Single	51.1	998
Married	17.0	332
Union / marital lives	24.0	469
Divorced / Separated	1.8	36
Widowed	6.0	118
Level of education		
Illiterate (never went to school)	14.4	281
Primary (1st to 6th grade)	47.7	931
Secondary (7th to 12th year)	32.9	642
Higher	5.1	99
Number of people working		
0	10.6	207
1	43.2	844
2	33.7	658
3+	12.5	244
Family Income (ECV)		
Less than 10,000	28.4	555
10,000 to 25,000	44.1	862
25,000 to 50,000	15.7	307
50,000 to 75,000	4.4	85
Greater than 75,000	2.7	53
N/A	4.7	91
Total	100.0	1953

*ECV* Cape Verdean escudos (1 EUR = 110.265 ECV), *N/A* Data not available

### Information about malaria

Malaria was the fifth (5 in 6), disease identified in the study communities (8, and all the respondents have already heard about it. Moreover, the majority of the respondents (85.3%) knew that malaria is transmitted through mosquito bites. Analysis by age showed that the level of knowledge about malaria transmission varies between different age range, being the highest in people with ≥41 years old (Table 3). About 96% of the respondents were aware that all the population is at risk of contracting malaria.

**Table 3:** The level of knowledge of the Cape Verdean population about malaria transmission by sex and age (%)

Characteristics	Mosquito bites	Contact with people with malaria	Contaminated food	Flies	Poor personal hygiene	Others	n
Age range							
≤ 29	22.0	0.1	0.3	0.2	0.7	1.3	495
30–39	19.3	0.4	0.1	0.1	0.9	0.5	412
≥ 40	44.0	0.4	0.8	0.7	3.4	2.4	1046
Sex							
Male	87.5	0.7	0.4	0.4	5.5	3.5	546
Female	84.4	0.6	1.4	1.1	4.8	4.4	1407
Total	85.3	0.8	1.1	0.9	5.0	4.1	1953

**Fig. 2:**
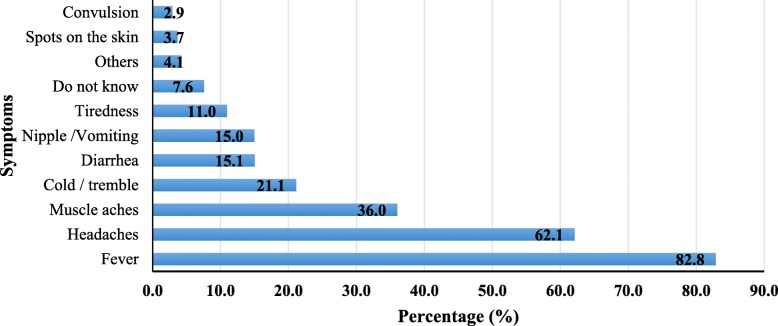
Main malaria symptoms known by the study population

The principal malaria symptoms identified in the study were fever (83%), headaches (62%) and muscle pains (36%) (Fig. 2).

### Treatment seeking behaviour

**Fig. 3:**
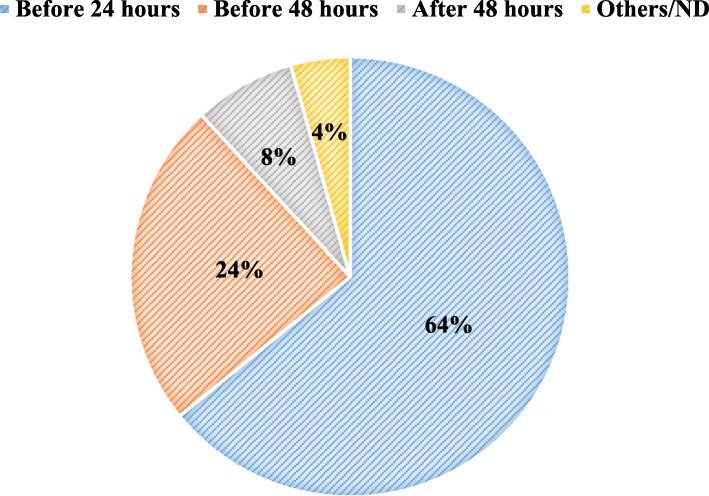
Time after which the cape verdean population see the health facility after the symptoms (of the fever), in percentage (%)

The population behaviour when facing malaria symptoms was analysed and revealed that 58% of the interviewed people seek for treatment at the nearest health structure, while 39% rely on self-medication and 3% seek for traditional methods of medication. The majority of people (64%) visit health structures or seek for treatment within the 24 h after the occurrence of first suspected malaria symptoms, against 24% that do it between 24 and 48 h, and 4% after 48 h (Fig. 3). However, when questioned about the best attitudes when facing malaria symptoms, 91% of the respondent’s reply “to immediately go to the nearest health structure”. In general, 86% of the people knew that delayed treatment seeking as well as delayed and inadequate treatment of malaria can lead to death or sequels.

Despite being yet underused in Cape Verde, insecticide-impregnated mosquito nets acceptability among the study population revealed that 97% of them have heard about mosquito nets, but only 19% were using them. However, 91% are open to the use if made freely available, against 5% who won’t and 4% who don’t know.

When asked who is in charge for malaria control in the community, 48% of the interviewed replied: “the population”, 33% “the health structure”, 13% “the government” and 18% thinks that “all the above” are. However, 3% of the respondents have designed other entities than the three above.

People perceive that coils (53%), spraying houses (45%), using repellent on exposed body parts (29%) or burying rubbish and sleeping underneath mosquito net (15%) protect against mosquito bites. Regarding which actions is considered the most important for a community-based malaria control, 53% of the interviewed indicated the involvement of all the country population, or encourage the community participation (32.4%), the participation of the media (13.5%), private sector (4.3%), church and pastors (3.7%) and 0.9% had no idea.

Questioned about the behaviour adopted when receiving a visit from health professionals, 96.2% said they always receive them, but 0.5% of the study population asked them to come back later and 0.3% do not receive them because of another commitment, while 1.3% claim another reason and 1.7% preferred to not respond to that question. The assessment of population knowledge about IRS activities in the country showed that 88.6% of them have had their house sprayed previously, against 11% who never benefited from the intervention, and 0.4% preferred not to respond. The majority of the population (95.8%) knew that IRS is beneficial for controlling mosquitos against 0.5% who didn’t think so, while 0.7% did not know and 3.0% did not respond.

### Sources of information about malaria

**Fig. 4:**
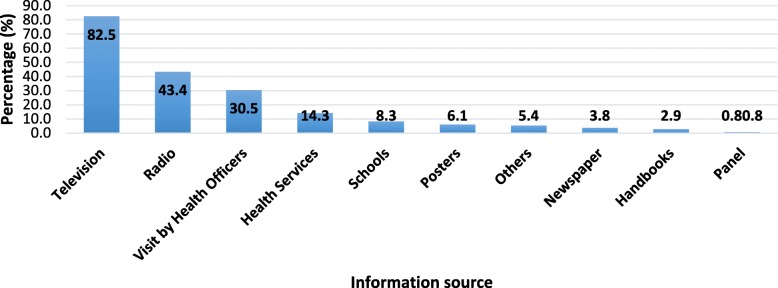
Source information about malaria in Cabo Verde

The main sources of information about malaria, including transmission, main symptoms and preventive measures were analysed. The results indicate that 90.4% of respondents received information about malaria, and the media are major sources of information. Being the television the principal medium (83%) followed by radio (43%). Home visit by health technicians (30%) was designed as other source of information (Fig. 4).

**Fig. 5:**
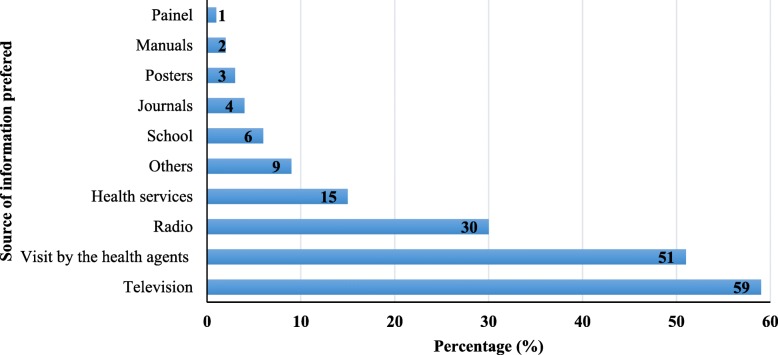
The preferred source of information about malaria for the Cape Verdean population

Overall, the majority of the population (89.2%) has a good understanding of information received about malaria, and only a few (6.7%) claimed the contrary. Among those having not understood the information, some reasons were appointed as, little information provided (24%), lack of adapted information for lay people (19%), not having a television (15%), not listening radio (8%), or lack of understanding of the technical language (8%), or other imprecise reasons (18%). When asked which ways they would like to receive information about malaria, the majority (59%) prefers being informed through the television and visits of health workers (51%) (Fig. 5).

When asked in which language they prefer being informed about malaria, the majority (86%) answered the Cape Verdean Creole, while 10% of the population preferred the Portuguese and 3% in both. Other preferred languages were English or French (1% each).

### Complete knowledge about malaria

We also assessed the overall level of the complete knowledge of the study population about malaria, including all the aspect of the disease transmission routes, at least one of the main symptoms of the disease and one preventive measure of the disease. The results show that 54% of the population had complete knowledge about the disease, being higher among men (60.3%) than women (52%). On the other hand, the complete knowledge was higher within the age group 35–39 years (60.7%), and within people with a higher level of education (75.8%) (Table 4).

**Table 4:** The complete knowledge about malaria among the different population groups (sex. age and scholar level)

Characteristics	n (total)	Percentage %)
Sex		
Male	329 (546)	60.3
Female	731 (1407)	52.0
Age group		
≤ 29	275 (495)	55.6
30–39	250 (412)	60.7
≥ 40	535 (1046)	51.1
Scholar level		
Analphabetic	98 (281)	34.9
Primary	485 (931)	52.1
Secondary	402 (642)	62.6
Higher	75 (99)	75.8
TOTAL	1060 (1953)	54.3

**Fig. 6:**
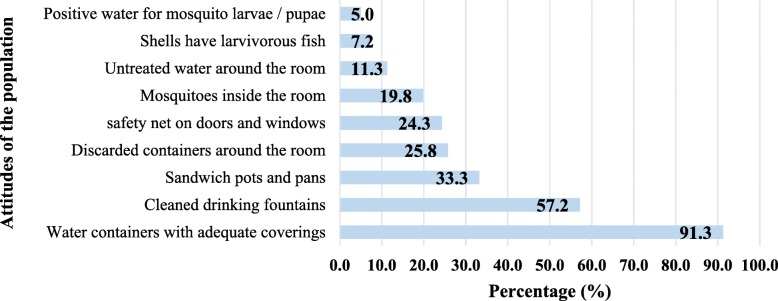
The Cape Verdean population attitudes regarding the malaria vector control tools

The visits of dwelling after each interview has allowed to cross-check and complete as much as possible the questionnaire. During the visits, the main findings of the population behaviours against mosquitoes were the adequate coverage of water containers (in 91% of visited houses), cleanness of animal’s troughs (57%), and fewer plants pots with sand (33%) (Fig. 6).

### Multivariate logistic regression

Using the logistic regression model [11], the following factors were associated with the complete knowledge about malaria (Table 5). When analysed, the scholar level (AOR 1.15; CI 1.0 to 1.0; *p* = 0.02), the resident area, in this case municipality (AOR = 0.9; CI 0.6 to 0.8; *p* = 0.00) and the age (AOR = 0.7; CI 0.6 to 0.2; p = 0.00) are the determinants that more influence the complete knowledge of population (Table 5).

**Table 5:** Logistic regression on age, resident area and scholar level and the prediction on complete knowledge about malaria in the capeverdian population

Covariates and analysed sample	Total	Univariate Analysis	Multivariate analysis
	Number (%)	Crude OR^a^ (95% CI)	AOR^a^ (95% CI)
Age (*n* = 1953)	1060 (54.3%)	1.12 (1.01–1.25)	0.71 (0,61 – 0,82)
Resident area (municipality) (1953)	1060 (54.3%)	1.00 (0.99–1.01)	0.99 (0.99–1.00)
Scholar level	1060 (54.3)	1.16 (1.03–1.30)	1.15 (1.02–1.69)

^a^*OR* Odds Ratio, *AOR* Adjusted Odds Ratio

## Discussion

The notable advances against malaria combined with scientific advances, had renewed the hope for malaria elimination in several WHO regions, one of which is Cape Verde. Nevertheless, to achieve the elimination goal and prevent resurgences of the disease, it is critical to implement strong and effective surveillance systems, to successfully and durably stop the transmission by detecting all possible malaria infections in the area in a timely manner [18]. This is where the important role of community involvement and the practices of the populations, especially those most affected by the disease [9, 10].

This first study conducted in Cabo Verde to explore the different levels of knowledge of the population about malaria at the national level. The population besides to know the correct form of malaria transmission and main symptoms, also know that the entire population is at risk of being affected by the disease. The attitudes to seek for treatment to the nearest health structure upon the apparition of the symptoms is in according with the WHO recommendation. Despite the low use of mosquito nets, the population has heard about it and is available to use it, in conjugation with other preventive measures. The population received information about malaria from media, mainly the TV and the radio and they e the complete knowledge of the disease.

From 2010 to 2016, Cabo Verde has recorded a few cases of malaria [6], is considered by the WHO as potentially able to eliminate malaria by 2020. However, an increase in 2016 and the outbreak recorded in 2017 with 446 cases [7], demonstrated weaknesses and challenges in the disease control.

Changes in the clinical epidemiology of malaria, such as the prevalence of asymptomatic carriers, changes in the spatial distribution of the disease becoming heterogeneous, with more local/focal and imported cases, emphasize the need to plan operationally viable strategies to timely and successfully identify all the reservoirs of parasites across all the residual malaria transmission areas [19]. Given the lack of up to date of the Cape Verdean knowledge, attitudes and practices about Malaria a KAP survey has been carried out to gather information to support the Elimination program, to better targeted and adapted interventions, as well as increase targeted population ownership and participation and acceptance for/of current and future Public Health program. These aspects are considered of extreme significance in countries and territories with low endemicity of the disease, especially in Cape Verde, an archipelagic country, where the distribution of cases is not geographically uniform [20, 21].

### Knowledge about malaria

Malaria has been classified as the fifth of the six major diseases in the country, which is likely related to few malaria cases recorded in the country, between 2010 and 2016 compared to other countries in the sub-region [6, 7, 22–25]. Therefore, the Cape Verdean population, excepted in the islands of Santiago and Boavista, are less in contact with malaria in their daily lives, thus losing their knowledge about malaria. On the other hand, the few numbers of malaria imported cases, exclusively from endemic countries of Africa, mostly asymptomatic [22, 23], makes it difficult to manage them in the country.

Mosquito bite has been identified as the principal malaria transmission as shown in some studies in Africa, Asia and America [26–36]. However, our results were in contrast with those previously reported from Nigeria, where a small proportion of respondents correctly know the main malaria transmission route and its cause [37].

The Cape Verdean population is aware that the entire national population are susceptible to the disease and has correctly identified mains malaria symptoms. Fever was reported as the most common symptom of the disease, together with headache, vomiting, chill and muscle pain. This is consistent with what was reported previously [37–39]. This high level of awareness about the disease, the modes of transmission and the clinical characteristics of malaria is likely to be related to the increased access to mass media, health education by health professionals, easy access of health services, as well as the reinforcement of door-to-door awareness campaigns carried out in recent years in Cape Verde [40].

### Aptitudes and practices of cape Verdean population toward malaria

The study showed that the majority of Cape Verdean looks for treatment to the nearest health structure, suggesting a good coverage of and accessibility to health facilities across the country [41]. The same has been concluded in African countries [31, 32, 42, 43], with the majority of studies relating high malaria treatment-seeking behaviour at health facilities with the availability and easy access of the latter. Conversely, this was not the case in Nigeria, where 47.6% of the population rely on self-medication but not health structures to treat malaria [37].

About 64% of the Cape Verdean population, go to the nearest health service within the 24 h upon the apparition of the first malaria symptoms, even more (88%) seek for treatment at health structures within the 48 h. This is in line with the Abuja summit report on malaria, stating that at least 60% of people suffering from malaria should seek treatment within 24 h of the apparition of first symptoms [44]. Despite the good treatment-seeking behaviour of the Cape Verdean population, there still more to do to get the closest to 100% of patients timely seeking treatment at the closest health facilities. Indeed, according to the WHO [45], the early search for treatment at the health structure is essential to interrupt the local transmission cycle of malaria, especially during the elimination phase. At this stage, the surveillance system should be able to detect all malaria infections and ensure radical treatment. Therefore, the awareness and collaboration of the population to directing themselves timely toward health structures earlier upon the apparition of malaria symptoms are crucial.

The results reported here are critical and encouraging in the context of the Cape Verdean malaria elimination programme, indeed, the study population displays a high level of knowledge about malaria symptoms and people know that the best behaviour when they are sick is to immediately seek for treatment at the nearest health structure. This knowledge must be transformed into routine attitudes and practices all over the country [46] to early detect all malaria infections, as required in malaria elimination strategies in the country [41] and the WHO elimination policies [42, 43, 47].

While in some African countries, there is a strong tradition of using mosquito bed nets against malaria [48–53], this strategy is not very common Cabo Verde [40]. However, the population awareness about the bed nets and their willingness to use them if easily available will facilitate their diffusion in the country. In fact, ITNs are widely accepted and used in several African countries, even in areas with no previous or low experience about the tool [42, 54]. Despite being influenced by seasonal factors, preferences criteria and cost, nets have been and still one of the cores if not the unique vector control strategy in some Africa’s settings with high availability (99% of households having at least one net). Nets are being mainly (76%) used throughout the year and, mainly during seasons of high densities of mosquitoes. In Cabo Verde, the low use may be associated with the non-adoption of this strategy by the National Malaria Control Program (NMCP) over the last years. The archipelago is not the unique case where low usage of mosquito nets against malaria has been reported. In South Africa, nets usage does not exceed 2% [42]. However, it is worth to note that despite being not implemented as a national malaria control strategy, The Cape Verdean NMCP is being gradually introducing and encouraging net usage as a personal protection measure with free distribution to all confirmed and hospitalized malaria patients, especially in places of high transmission risk.

Despite the efforts made so far to malaria control in Cabo Verde, social awareness in some aspects of the disease still need to be strengthened. This is particularly true when asking about who should be the stakeholders of mosquito’s control within the community. Most people think that that the population alone or health structures alone are responsible for the actions to be taken, while only a small part believe both should joint their effort to control vector, and thus the diseases they transmit. On the other hand, most respondents believe that malaria is preventable, but few of them still thinking that there no need for personal protection against infection or opt for other non-conventional strategies. Therefore, the issue of not combating malaria may be associated with the people dependence on local health structures for malaria control interventions. Moreover, the lack of knowledge of the most correct actions needs to be further studied.

Interviewees have a good knowledge of environmental actions as good preventive measures, among which the main measures consisted for them to eliminate breeding sites at the community level. This has been revealed in other studies in Nigeria [55] and Ethiopia [56], where the study population saw environmental management as an efficacious mosquitos control strategy. However, knowledge has not always been translated into the improved practice of preventive measures. Therefore, targeted and adapted actions should be developed to empower the Cape Verdean populations for best practices, given the low socioeconomic level and the low level of formal education of rural communities.

IRS is one of the main vector-borne diseases control strategies, especially against malaria [57]. Most of the participant’s knowledge IRS importance to control mosquitoes and consequently malaria. However, only 42.9% of households were sprayed within the last 12 months preceding the survey. This is lesser than the WHO recommended minimum IRS coverage rate of 80% for community protection [56]. Unfortunately, the same has been reported from several other countries [28, 42, 56, 58]. But, countries such as the Swaziland where IRS coverage was above 80%, shows that the goal could be achieved with the increase of the commitment of local stakeholder and community [35].

### Source of information on malaria

The television, radio as well as community sensitisation by health agent’s visits, have been identified most as main sources of information about malaria in Cape Verde as shown in South Africa [42]. In contrast, in Swaziland information about malaria were mainly retrieved from health facilities in 2009 [35]. Our results have revealed that most of the Cape Verdeans are well informed about malaria and understand well the information they receive. Which is very interesting, given the malaria low incidence in the country. In fact, not being affected by the disease may contribute to the decrease of the awareness about it. Moreover, only hearing about malaria is not enough but should be the basis addressing a whole range of issues about the disease, especially to increase knowledge about the form of transmission, the main symptoms, preventive measures and treatment.

Besides the conventional media such as TV and radio, technological advances offer new avenues to increase the population awareness about malaria-associated issues in Cabo Verde. Indeed, phone and SMS, as well as new social media platforms, should not be ignored as potential and very accessible source of information. This is particularly true concerning the specificities of each island regarding the educational level of the population; and despite the majority of the population prefer being informed through television or visits by health workers and mainly in Creole, their local language.

### Complete knowledge of malaria

In summary, the complete local population knowledge about malaria, including transmission routes, symptoms and main preventive measure showed that more than half of people know enough about malaria, which is strongly correlated with the level educational. Therefore, the relatively high frequency of illiteracy among the participants (14.4%) plays a key role in limiting the acquisition of knowledge or awareness about malaria. This is very important in a context where malaria was no longer a huge public health problem in the country, until the recent outbreak [6].

This study also revealed significant differences in protective measure against mosquito bites, such as the use of mosquito nets and sprays cans to prevent malaria transmission. Therefore, it is urgently needed to promote equal as well as universal access and usage of malaria preventive measures to get back on track of the malaria elimination goal in Cabo Verde, especially in Santiago and Boavista islands where all the malaria indigenous cases were in 2017 [6]. This is also true to prevent the re-introduction of malaria once the archipelago would be declared malaria-free.

To that aim, great importance must be given to behaviour change communication (BCC) strategic using targeted messages and personalized approaches to promote healthy behaviours and reduce risks [59]. In Cabo Verde, BCC mechanisms and community involvement, using the new tools and strategies for malaria elimination still a big challenge [19], as shown by the local population participation in mosquito control activities. Indeed, only less than 40% of the study population reported having previously participated in some vector control activities. Strong evidence exist elsewhere on the key role that must be played by BBC to improving malaria control and prevention as well as treatment-seeking behaviours [60–64]. For instance, the promotion of screening and treatment of malaria in hotspots areas in Zanzibar or the use of nets and prophylaxis for travellers in Swaziland [65], have led to a significant decrease of malaria incidence. Other aspects for which BCC is fundamental concern the screening and detection of asymptomatic individuals requiring some time mass diagnosis and treatment of as much as possible individuals or communities [66]. These two essential aspects should be reinforced in Cabo Verde, considering the circumscription of indigenous malaria cases in well-identified hotspot locations.

The country-level Malaria Communication Strategic Framework [67] argues that well-planned and well-implemented communication programs can contribute to achieving multi-level malaria prevention and treatment targets. These findings of this study demonstrate that the behavioural change communication strategies should be updated and adapted to the changing profile of the disease transmission and the risk of transmission to achieve the elimination in Cabo Verde. Also, timely studies should be implemented frequently to gather evidence to adapt messages and approaches, reduce audience scorn, promote new interventions and solidify the evidence base that allows adapting efforts to respond to demands on the process of malaria in Cabo Verde.

## Conclusion

Overall, the study revealed that the Cape Verdean population has a high of knowledge about malaria, including its mode of transmission, main symptoms, adequate behaviours towards its treatment and main preventive measures. However, some gaps and misunderstandings persist about the disease, being strongly correlated to the level of education.

The population usually seek for treatment to the nearest health structure within the first 24 or 48 h after the apparition of main malaria symptoms. However, attitudes and practices regarding preventive measure need to be improved. Some barriers make the control actions unsatisfactory and undesirable, reflected by ignorance and/or indifference of the targeted population about malaria prevention. Adapting strategies to increase knowledge of the population, including the malaria transmission, the benefits of preventive measures and the availability of effective individual and community control tools, need to be strengthened the malaria elimination process in Cabo Verde.

## Additional files

Additional file 1: Interview Guide used in KAP Study (DOCX 27 kb)

## Data Availability

We did not obtain consent to share data obtained from the questionnaire and key informant interviews, however the datasets used and/or analysed during the current study available from the corresponding author on reasonable request.

## Abbreviations

BCC: Behaviour Change Communication
CNEPS: Comité Nacional de Ética e Pesquisas em Saúde
IRS: Indoor Residual Spray
ITNs: Insecticide-treated bed nets
KAP: Knowledge, attitudes and practices
NMCP: Nacional Malaria Control Program
SPSS: Statistical Package for the Social Sciences
SQD: Squares of distances
WHO: World Health Organisation
