# Supplementary material for: Knowledge, attitudes and practices about malaria in Cabo Verde: a country in the pre-elimination context
Source: BMC Public Health. 2019 Jul 1;19:850. doi: 10.1186/s12889-019-7130-5 (PMC6604228; doi:10.1186/s12889-019-7130-5)
Supplement: Supplementary file 1 — Interview Guide used in KAP Study (DOCX 27 kb) [file 12889_2019_7130_MOESM1_ESM.docx]

## Additional File 1 - Interview Guide used in KAP Study

| **IDENTIFICATION** | |
| --- | --- |
| DATE____ /____/______ QUESTIONNAIRE NUMBER: __________________ | |
| ISLAND___________________________________________ | MUNICIPALITY:___________________________________ |
| COMUNITY: ____________________________________ | NAME OF INTERVIEW LOCATION: ______________ |
| INQUIRER /A:___________________________________________ | SUPERVISOR: ___________________________________ |
| Good Morning /Afternoon. My name is _________________________. I am working for the National Directorate of Health through the National Program to Combat vector-borne diseases and Health Problems related to the Environment and CCS-SIDA, in a research on "KNOWLEDGE OF KNOWLEDGE, ATTITUDE AND PRACTICE, POPULATION IN RELATIONSHIP TO MALARIA AND ITS PREVENTION ", with the aim of collecting information for the development of a strategy in order to minimize possible negative impacts and maximize the positive ones with this action.  Any information you provide will be kept confidential and will only serve the purposes of the research. Your participation is voluntary and anonymous, and you can decide to answer any or all of the questions. However, we hope you will participate in this research because your contribution is very important to the Study.  Do you want to ask about the research?  Can I start the questions? | |

| **A.**  **DEMOGRAPHIC CHARACTERISTICS** | | | | | | | | | |
| --- | --- | --- | --- | --- | --- | --- | --- | --- | --- |
| **A.1**  **How many full years do you have** ? [____!____] Years | | | | **A.2 What is your gender ?** | | | | M | F |
| **A.3 What is your qualification ?**  [1] illiterate [never went to school]  [2] primary [1st to 6th grade]  [3] Secondary [7th to 12th Year]  [4] Top / University  **A.4. Nationality**  [1] cape Verdean [2] other: ___________________________________ | | | | **A.5 What is your marital status ?**  [1] Single  [2] Married  [3] Committed / marital lives  [4] Divorced [5] Separated  [6] Widowed | | | | | |
| **A.6** | **A.7** | **A.8** | **A. 9** | **A.10** | | | **A.11** | **A.12** | **A.13** |
| Line No. | No. Number of usual residents or visitors | Relation with the head of the household  [1] Husband  [2] Wife  [3] Child  [4] Nephew  [5] Neto  [6] Godson  [7] Other | Sex  [1] Male  [2] Female | Residence | | | How old is?  [Please indicate age in years] | Woman of 15-49 years eligible (circle the line number)  [1] Yes  [2] No | She is pregnant?  [1] Yes  [2] No  [3] N/A |
|  |  |  |  | Does he (she) live here?  [1] Yes  [2] No | | Did not he sleep here last night?  [1] Yes  [2] No |  |  |  |
| 01 | [___] | [___] | [___] | [___] | | | [__!__] | [___] | [___] |
| 02 | [___] | [___] | [___] | [___] | | | [__!__] | [___] | [___] |
| 03 | [___] | [___] | [___] | [___] | | | [__!__] | [___] | [___] |
| 04 | [___] | [___] | [___] | [___] | | | [__!__] | [___] | [___] |
| 05 | [___] | [___] | [___] | [___] | | | [__!__] | [___] | [___] |
| 06 | [___] | [___] | [___] | [___] | | | [__!__] | [___] | [___] |
| 07 | [___] | [___] | [___] | [___] | | | [__!__] | [___] | [___] |
| 08 | [___] | [___] | [___] | [___] | | | [__!__] | [___] | [___] |
| **B**  **SOCIODEMOGRAPHIC DATA** | | | | | | | | | |
| **B.1** **What is your profession:**  [1] Primary Sector  [2] Secondary Sector  [3] Tertiary Sector  [4] Other: _________________________  [5] Anything | | | | **B. 2** **How many people work in this house ?**  ________ People | | | | | |
| **B.3 What is the family income?**  [1] <10,000 $ 00  [2] 10,000 $ 00 to $ 25,000  [3] $ 25,000 to $ 50,000  [4] $ 50,000 to $ 75,000  [5]> 75,000 $ 000 | | | | **B.4 -**  **How many rooms have your home ?**  **_____________**  **B.5 - Your home is:**  [1] own  [2] rented  [3] other: ________________________________ | | | | | |
| **B. 6 –**  **Access to the sewage system**  [1] Yes  [2] No | | | | **B. 7 -**  **Access to Water**  [1] standpipes    [2] Fountains    [3] pump trucks     [4] supply network    [5] Other: ______ | | | | | |
|  | | | |  | | | | | |
| **C.**  **KNOWLEDGE ON MALARIA** | | | | | | | | | |
| **C.1-**  **What are the most common diseases in this community?**       [1] Malaria        [2] Diarrhea     [3] Intestinal parasites       [4] Other _____________________ | | | | **C.2 -**  **Have you heard about malaria?**  [1] Yes  [2] No | | | | | |
| **C.3 -**  **How is Malaria Transmitted?**  [1] Contact with people with the disease  [2] Contaminated food [3] Mosquito bites  [4] Through Flies  [5] Poor personal hygiene  [6] Other: _____________________ | | | | **C.4 -**  **Who can contract malaria?**  [1] Children [2] Youth  [3] Adults [4] Men  [5] Women [6] All | | | | | |
| **C.5 -**  **If YES, what are the signs and symptoms of malaria you know?**  [1] fever [2] Headaches  [3] Muscle pain [4] Convulsion  [5] Diarrhea [6] skin blemishes  [7] Headache [8] Nausea  [9] Cold / Fear [10] Tiredness  [11] Do not know [12] Other ______________ | | | | **C.6 -**  **In the last 12 months, did anyone in this household have malaria?**  [1] Yes  [2] No  [3] Do not Know | | | | | |
| **C.7 -**  **If YES, how many people?**  ________ People. | | | | **C.8-**  **Has anyone in your family had a fever in the last 6 months?**  1] Yes [2] No | | | | | |
| **D.**  **PROCEDURE IN CASE OF DISEASE / CASE MANAGEMENT** | | | | | | | | | |
| **D.1 -**  **What should a person do when developing malaria symptoms?**  [1] self-medicate [2] Go to the post health  [3] Go to the Health Center [4] Go to the Hospital  [5] Go private clinic [6] Nothing  [7] Other ________________________ | | | | **D.2**  **How long after the fever does the above-mentioned attitude take place?**            [1] Before 24 hours            [2] Before 48 hours            [3] After 48 hours | | | | | |
| **D. 3 - O que uma pessoa deve fazer ao desenvolver sintomas do paludismo?**  [1] Take some medication on your own  [2] Take tea  [3] Wait for me to improve spontaneously  [4] Move immediately to the nearest health structure  [5] Other: ______________________________ | | | | **D.4 –**  **What are the consequences of Malaria for your health?**  [1] The disease will pass by itself  [2] It does not hurt to health  [3] Can it get worse and lead to death?  [4] Can you leave sequels  [5] Other: ______________________________ | | | | | |
| E. **USING MOSQUITOES NETS** | | | | | | | | | |
| **E.1 -**  **Have you heard of Mosquito nets?**  [1] Yes [2] No | | | | **E.2 - Do you use Mosquito Netting?**  [1] Yes [2] No | | | | | |
| **E.3 -**  **Why not use it?**  [1] I do not know how useful [2] I do not have  [3] I do not know where to get [4] I do not like it, it gets ugly;  [5] Does Heat [6] Do not sit mosquitoes  [5] Other: ______________________________ | | | | **E.4 -**  **If a bednet is offered, would you use it?**  [1] Yes  [2] No  [3] Do not know | | | | | |
| **F.** **VECTOR CONTROL** | | | | | | | | | |
| **F.1 -**  **Is there mosquitoes in this community?**  [1] Yes [2] No  **F.2 –**  **Do you participate in cleaning and anti-malaria campaigns organized in the locality?**  [1] Yes [2] No | | | | | **F.3 -**  **What can be done to avoid mosquito bites?**  [1] Nothing [2] Making Smoke  [3] Spray House  [4] Using repellents on the exposed part of the body  [5] Burying trash    [6] Sleep low mosquito net    [7] Do not know    [8] Other _________________ | | | | |
| **F.4 - Who is the main responsibility in the fight against malaria?**  [1] Government  [2] Health Offices  [3] Town Councils  [4] Population  [5] Other: ______________________________ | | | | | **F.5**  **What have you done to avoid malaria?**  [1] How do you do it?  [2] Spraying houses with flits  [3] Remove / eliminate breeding grounds within and outside housing  [4] Other | | | | |
| **F.6 - What are the most important actions in the fight against malaria?**  [1] Community Involvement;  [2] Participation of the private sector  [3] Church involvement  [4] Social Communication Participation  [5] Anything  [6] All | | | | | **F.7 -**  **How has your behavior been when you visit a health professional?**  [1] You always receive it  [2] Ask to come back later or another day  [3] Do not receive it because of another commitment  [4] Other: __________________________ | | | | |
| **G.**  **INTRADOMICILIARY SPRAYING** | | | | | | | | | |
| **G.1 -**  **Do you know that you spray in the home in the whole country?**  [1] Yes [2] No | | | | | **G.2**  **If yes, have your home been sprayed in the last 12 months?**  [1] Yes When? (Month)___________________  [2] No | | | | |
| **G.3 -**  **If not, why?**  [1] Dirty wall  [2] causes itching  [3] I was not at home  [4] No effect  [5] I heard that the product does hurt people  [6] Others (please specify) ____________ | | | | | **G.4 - If Yes, do you think this action has benefits?**  [1] Yes  [2] No  [2] Do not know | | | | |
|  |  |  |  |  | **G.5 - Which benefits?**  [1] Reduces malaria  [2] Reduces number of bites by mosquitoes  [3] Do not know  [4] Others (please specify) | | | | |
| **H.** **MEANS OF OBTAINING INFORMATION ABOUT MALARIA** | | | | | | | | | |
| **H.1-**  **Have you received information about malaria?**  [1] Yes  [2] No  **H.3 -**  **Do you usually participate in malaria (in) training sessions?**  [1] Yes  [2] No | | | | | **H.2 - If yes, where do you get the information on malaria?**  [1] Radio [2] Television  [3] Schools [5] Posters  [6] Journal   [7] Manuals  [8] Health Services  [9] Other ______________________ | | | | |
| **H.4 – Do you understand past information about malaria?**  [1] Yes  [2] No | | | | | **H.5. If not, why?**  [1] Little information  [2] I do not understand the language  [3] I do not watch TV  [4] Do not listen to the radio  [5] I do not get information  [6] Other: ___________________________________ | | | | |
| **H.6 -**  **How would you like information about malaria?**  [1] Radio [2] Television  [3] Schools [5] Posters  [6] Journal   [7] Manuals  [8] Health Services  [9] Other ______________________ | | | | | **H.7 - In what language would you like to receive information about malaria?**  [1] Creole  [2] English  [3] Other: __________________ | | | | |
| **H.8 - Do you implement the recommendations received on malaria and other mosquito-borne diseases?**  [1] Yes [2] No [3] Other | | | | | **H.9 - Any suggestions for improving malaria control in Cape Verde?**  **__________________________________________________________** | | | | |

## 11.4 - Check list

| **Nº** | **Points to be observed** | | **YES** | **NO** | **OBS** |
| --- | --- | --- | --- | --- | --- |
| **1** | | Do doors and windows have a safety net? |  |  |  |
| **2** | | Are water containers adequately covered? |  |  |  |
| **3** | | Do the deposits have larvivorous fish? |  |  |  |
| **4** | | Existence of untreated water collections around the house? |  |  |  |
| **5** | | Do you see discarded containers around the room? |  |  |  |
| **6** | | The pots of the plant pots are filled with harness? |  |  |  |
| **7** | | Do you observe adult mosquitoes inside the house? |  |  |  |
| **8** | | Have you found at least one container with positive water for mosquito larvae or pupae? |  |  |  |
| **9** | | Are the animal drinkers clean? |  |  |  |
